# Supplementary material for: Differential Entropy: An Appropriate Analysis to Interpret the Shape Complexity of Self-Similar Organic Islands
Source: Materials (Basel). 2021 Oct 29;14(21):6529. doi: 10.3390/ma14216529 (PMC8585197; doi:10.3390/ma14216529)
Supplement: Supplementary file 1 [file materials-14-06529-s001.zip › materials-1402173-supplementary.pdf]

Supplementary materials

Differential Entropy: An Appropriate Analysis to Interpret the Shape Complexity of Self-Similar Organic Islands

Stefano Chiodini [1,2], Pablo Stoliar [3], Pablo F. Garrido [4] and Cristiano Albonetti [1,*]

1 Consiglio Nazionale delle Ricerche—Istituto per lo Studio dei Materiali Nanostrutturati (CNR-ISMN), Via P. Gobetti 101, 40129 Bologna, Italy; Stefano.Chiodini@iit.it
2 Center for Nano Science and Technology, Fondazione Istituto Italiano di Tecnologia, Via G. Pascoli 70, 20133 Milan, Italy
3 National Institute of Advanced Industrial Science and Technology (AIST), Tsukuba 305-8565, Ibaraki, Japan; p.stoliar@aist.go.jp
4 Departamento de Fisica de Aplicada, Facultade de Fisica, Universidade de Santiago de Compostela, E-15782 Santiago de Compostela, Spain; Pablo.Fernandez@usc.es
* Correspondence: cristiano.albonetti@cnr.it

Citation: Chiodini, S.; Stoliar, P.; Garrido, P.F.; Albonetti, C. Differential Entropy: An Appropriate Analysis to Interpret the Shape Complexity of Self-Similar Organic Islands. *Materials* **2021**, *14*, 6529. https://doi.org/10.3390/ma14216529

Academic Editor: Oleg Igorevich Lebedev

Received: 15 September 2021
Accepted: 25 October 2021
Published: 29 October 2021

Publisher's Note: MDPI stays neutral with regard to jurisdictional claims in published maps and institutional affiliations.

Copyright: © 2021 by the authors. Licensee MDPI, Basel, Switzerland. This article is an open access article distributed under the terms and conditions of the Creative Commons Attribution (CC BY) license (https://creativecommons.org/licenses/by/4.0/).

1. Fractal Dimension D_f Evaluated by the Box Counting Method

Topographic AFM images were processed with Gwyddion software [1]: (1) Data were levelled by the mean plane subtraction; (2) Paraboloidal background was removed; (3) Rows were aligned by means of the median of differences; (4) Horizontal scars were corrected; (5) Mean values filter (3 × 3 px^2) is applied to remove possible topographic artefacts. Once processed, AFM images of 6T islands are firstly marked by threshold, then selected islands are filtered for area in order to remove larger islands due to bimodal growth (see main text). The resulting mask is extracted and used to statistically evaluate the average area of islands (black islands in Figure S1), thus determining the largest box size (closeness power of 2) employed in the box counting method. As required for fractal calculus in ImageJ [2], the mask is changed to 8 bit and binarized, then fractal dimension is calculated by using power of 2 box sizes, i.e. 2^0, 2^1, 2^2,... (see Figure S1).

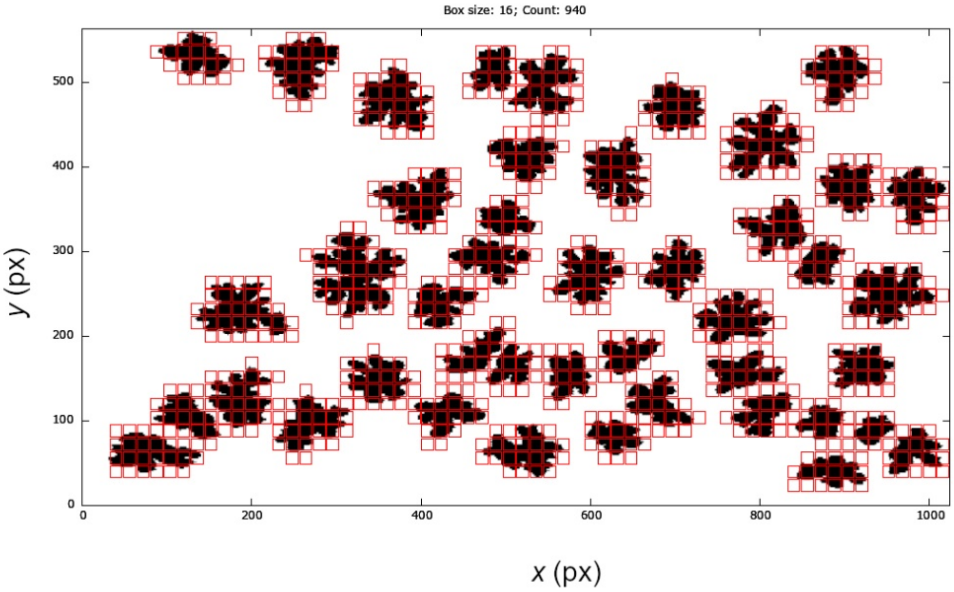

Figure S1. Illustrative example of the box counting method applied to an AFM image (1024 × 512 px^2) composed of 6T islands with average area of 2850 px^2, correspondent to a largest box size of

53 px that is closeness to a power of 2 box size of 64 px = 2^6 px. This example shows islands covering of boxes with size 16 px = 2^4 px.

2. Differential Entropy Definition

The differential entropy extends the Shannon entropy, a measure of average surprisal of a random variable [3], to a continuous probability distribution [4]. From Shannon information theory [3], (Shannon) entropy *SE* is defined as:

$$\mathrm{SE} = -\sum \mathrm{p_i} \log_2 \mathrm{p_i} \tag{S1}$$

In this formulation, *p* is the probability of a data point or "count" *c* to has a value within a specific data interval *i* (bin). Thus, *p* is calculated as:

$$\mathrm{p_i} = \frac{\mathrm{c_i}}{\sum \mathrm{c_i}} \tag{S2}$$

As illustrative example, histograms of Figure 1 in Reference [5] have 18 bins thus *SE* ranges from a minimum of 0 (top histogram, c_i has only a possible value hence p_i =1 and *SE* = 0 from Equation (S1) to a maximum of approximately 4.2 (bottom histogram, c_i is equal to all bins p_i =1/18—equiprobable events—and $SE = -18 \cdot 1/18 \cdot \log_2 (1/18) = -[\log_2 (1) - \log_2 (18)] = -[-\log_2 (18)] \sim 4.2$). The middle histogram shows a data distribution more typically with an *SE* value in between the two limits and, accordingly, maximum *SE* value depends on the number of bins selected for data representation. An *SE* value of zero, corresponding to a single bin probability of one, indicates that a descriptor adopts only one value and has thus no information content with respect to the data set. On the other hand, the maximum *SE* value will be observed if all possible descriptor values are equally probable, which corresponds to maximum information content. From Equation S1 the log function is taken to be $\log_2$, so the entropy *SE* is expressed in bits (dimensionless) [6].

By following the aforementioned description, *SE* limits for our angle distributions (taken the discrete version of Equation S1 in the main text and using a logarithm in base 2) are 0 and ~ 7.5, because of bins number has been consistently set to 180 with a bin size of $\Delta\alpha = 1°$.

By definition, for a continuous random variable x with probability density function $p(x)$, the differential entropy *S*, i.e. the continuous counterpart of Equation S1, is:

$$S(x) = -\int_{x_i}^{x_f} p(x) ln\big(p(x)\big) dx \tag{S3}$$

where the initial $x_i/p_i(x)$ and final $x_f/p_f(x)$ integral limits are > 0 [6]. As claimed by Marsh [4], "*the formula for continuous entropy is not a derivation of anything, unlike Shannon entropy, it's merely the result of replacing the summation with an integration*" although "*best of all, (it) allow us a means of justifying and proving results with the same familiar, intuitive feel granted us in the discrete realm*". From Equation S3 the log function is taken to be *ln*, so the differential entropy *S* is expressed in nats [6].

The limits of *S* from Equation S3 are 0 and, in case of equiprobable events with a constant $p(x) = a$, $S = -a \cdot ln(a) \cdot (x_f - x_i)$. Specifically, they are 0 and ~ 5.2 nats from Equation 1 of the main text.

3. Differential Entropy Computation

Next, we summarize the procedure to compute the differential entropy. The full technical details for each step are described in the next section. The first step to compute

the differential entropy was segmentation of the AFM images [7]. We developed an *ad hoc* image segmentation procedure that consists of three steps (see Figure S2).

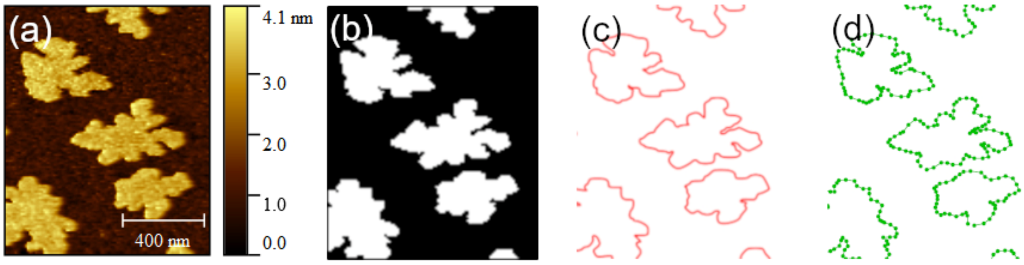

Figure S2. Steps to obtain polygonal chains describing the contour of the islands. (**a**) Original AFM image. (**b**) Two-levels bitmap image where islands -white- are clearly identified on the background -black-. (**c**) Contours of the islands traced as a series of lines and Bezier curves. (**d**) Contours described as polygonal chains (each dot is a vertex).

We processed the AFM image (Figure S2a) to create a two-levels bitmap image where islands–white–were clearly separated from the background–black–(Figure S2b). Then, we traced the contour of the island by using the "trace bitmap" function of the Inkscape software (Figure S2c) [8]. This function detects the contours into the bitmap image and "trace" them using a series of lines and cubic Bezier curves. Finally, we turned the traced contours into polygonal chains made up of relatively small segments (Figure S2d).

Then, angles were evaluated between each segment and a radial line connecting the middle of the segment and the centroid of the island (see Figure S3).

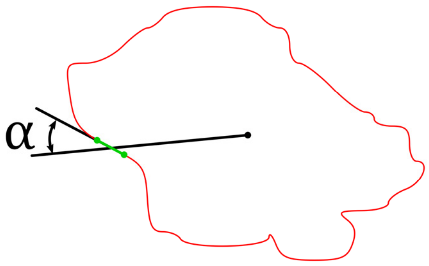

Figure S3. Angle α between a segment and the radial line to the centroid of the island (this image is only a qualitative hand-draw representation).

The probability density function, $p(\alpha)$, of the distribution of angles is then estimated. Figure S4 presents two examples of pdf for different island shapes. Compact islands show a distribution of angles with higher probability around 90° (Figure S4a,S4b) while dendritic islands have a uniform pdf (Figure S4c,S4d).

Finally, based on these probability density functions, we computed the differential entropy as:

$$S = -\int_0^{180} p(\alpha)\, ln[p(\alpha)]\, d\alpha \quad \text{(S4)}$$

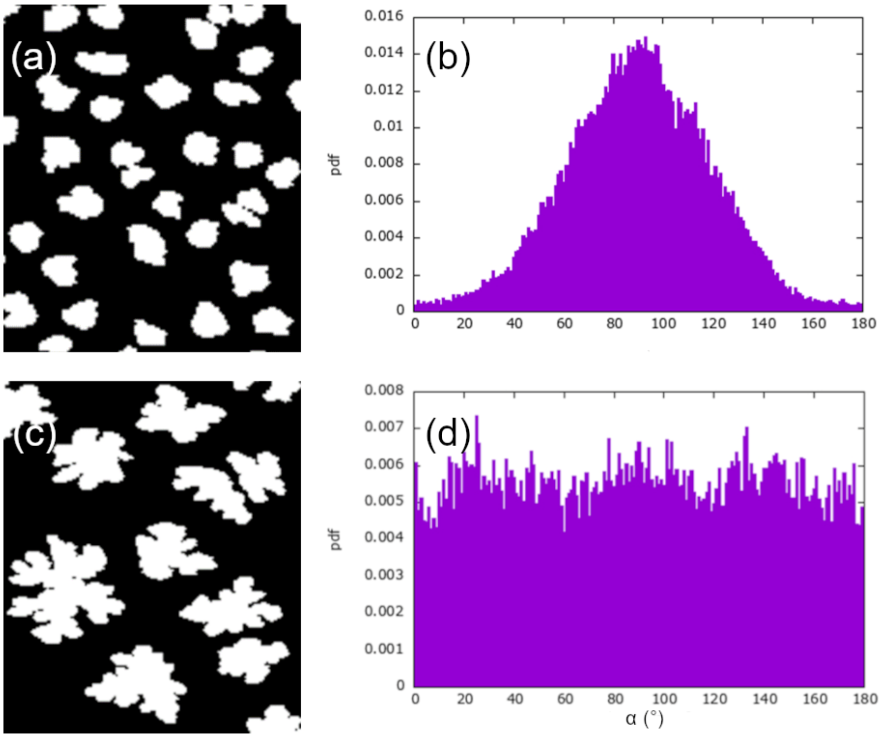

Figure S4. Probability density functions of the angles α distribution for compact (**a**,**b**) and dendritic islands (**c**,**d**).

4. Technical Details on Images Processing

4.1. AFM Image Processing to Obtain Two-level Bitmaps

Topographic AFM images were processed with Gwyddion software [1]: (1) Data were levelled by the mean plane subtraction; (2) Paraboloidal background was removed; (3) Rows were aligned by means of the median of differences; (4) Horizontal scars were corrected; (5) Mean values filter (3×3 px^2) is applied to remove possible topographic artefacts. Then, 6T islands are marked by threshold, and selected islands are filtered for area in order to remove larger islands induced by bimodal growth. The resulting mask is extracted, obtaining a two-level bitmap with black background and white islands (see main text).

4.2. Turning Islands Contours into Polygonal Chains

The bitmap image is traced by using Inkscape software [8]. The "DPI" settings of the BMP file were firstly cleared, then imported in Inkscape software by using the following setting: image import type = embed, image DPI = "default import resolution", image rendering mode = none.

The "trace bitmap" function runs with the following settings: Single scan, Brightness cut-off mode, brightness threshold = 0.45, Invert image, speckles = 2, smooth corners = 1, optimize = 0.2. Figure S5 shows the original two-levels bitmap (black and white) imported in Inkscape with traced contours (red).

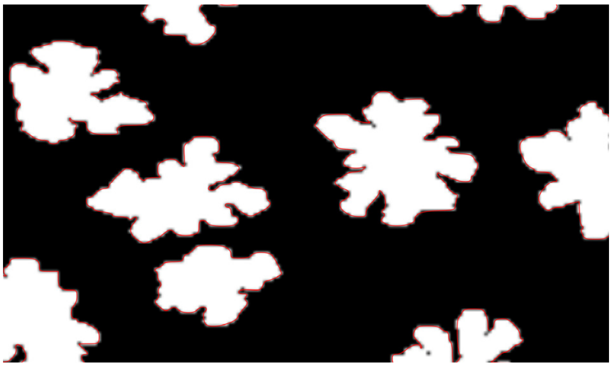

Figure S5. Inkscape screenshot showing islands (white) of the imported bitmap image with traced contours (red lines).

The file was saved in SVG format and it was processed with *ad hoc* and home-developed Python script (see next Section 4). Such script first reads islands contours stored into the SVG file. Specifically, the contours are stored as a sequence of drawing commands, each drawing a segment [9]. These commands basically draw lines or Bezier curves (also arcs, but they were never present in actual AFM images). Each island in the AFM image is separated by a "close path" command. The lines were divided into segments of length $\Delta l = 0.1px$ (i.e., ~10 times oversampling). The Bezier curves were also divided into segments with roughly the same Δl. Nevertheless, in the case of this curves, the segments were not all equal length; we performed a rough estimate of the curve length, L, as:

$$L \approx |P_3 - P_2| + |P_2 - P_1| + |P_1 - P_0| \tag{S5}$$

where $P_0 \ldots P_3$ are the control points [10]. Then, the total number of segments as ~$L/\Delta l$ were estimated and used in the explicit formula of the cubic Bezier curve for computing vertices of segments [10]:

$$X(t) = (1-t)^3 P_0 + 3(1-t)^2 t P_1 + 3(1-t)t^2 P_2 + t^3 P_3 \tag{S6}$$

where *t* ranges from 0 to 1 in $L/\Delta l$ steps.

4.3. Islands Centroids

The algorithm described in Reference [11] was used to compute the centroid of the polygon, (C_x, C_y):

$$C_x = \frac{1}{6A}\sum_i (x_i + x_{i+1})(x_i y_{i+1} - x_{i+1} y_i) \tag{S7}$$

$$C_y = \frac{1}{6A}\sum_i (y_i + y_{i+1})(x_i y_{i+1} - x_{i+1} y_i)$$

where (x_i, y_i) are the vertices of the polygon, and *A* is the area, computed as:

$$A = \frac{1}{2}\sum_i (x_i y_{i+1} - x_{i+1} y_i) \tag{S8}$$

4.4. Probability Density Function and Differential Entropy Calculi

The pdf, $p(\alpha)$, was estimated as a piecewise function divided into $N = 180$ intervals of $\Delta\alpha = 1°$ each. The *n*-th interval, comprised between $n \cdot \Delta\alpha \leq \alpha \leq (n+1) \cdot \Delta\alpha$ for $n = 0 \ldots N\text{-}1$, is computed as:

$$p(n) = \frac{\sum_j \begin{cases} l_j & n\,\Delta\alpha \le \alpha < (n+1)\,\Delta\alpha \\ 0 & otherwise \end{cases}}{\Delta\alpha\,\sum_j l_j} \quad \text{(S9)}$$

where l is the length of the segment, and j iterates along all the segments of all the polygons (islands) of the image. The area of each interval $p(n)\cdot\Delta\alpha$ each interval is the probability of having an angle α comprised within the limits of the interval.

Lastly, the differential entropy is calculated as:

$$\mathrm{S} = -\Delta\alpha \sum_{\mathrm{n}} \mathrm{p(n)}\ln[\mathrm{p(n)}] \quad \text{(S10)}$$

5. Python Script

Python code is available in the website https://github.com/Cristiano1974a/Python_code.git and distributed with GNU General Public License v3.0 (accessed on August 25th 2021).

6. Linear Decrease of Slopes vs Θ

The trend of D_f vs Θ slopes, δ (in ML^{-1}), is independent to T_S because δ decrease linearly with the same slope, i.e. -0.14 ML^{-2}. Obviously, D_f at 50 °C is larger than at RT (see Figure S6).

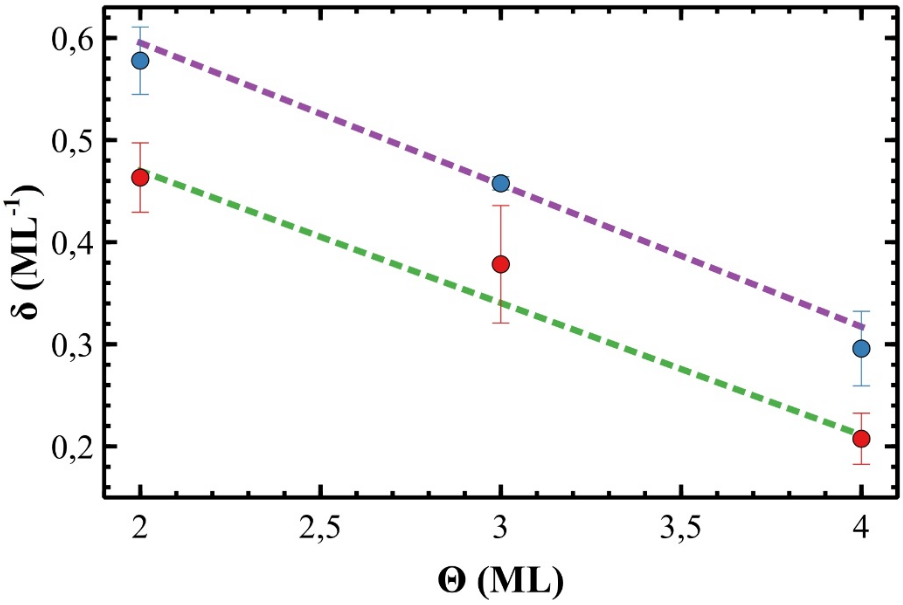

Figure S6. Linear decreasing of δ vs Θ (in ML) for RT (red dots) and 50 °C (blue dots). Linear fits have clearly the same slope.

7. Representative Islands Pdfs for Some Growth Steps

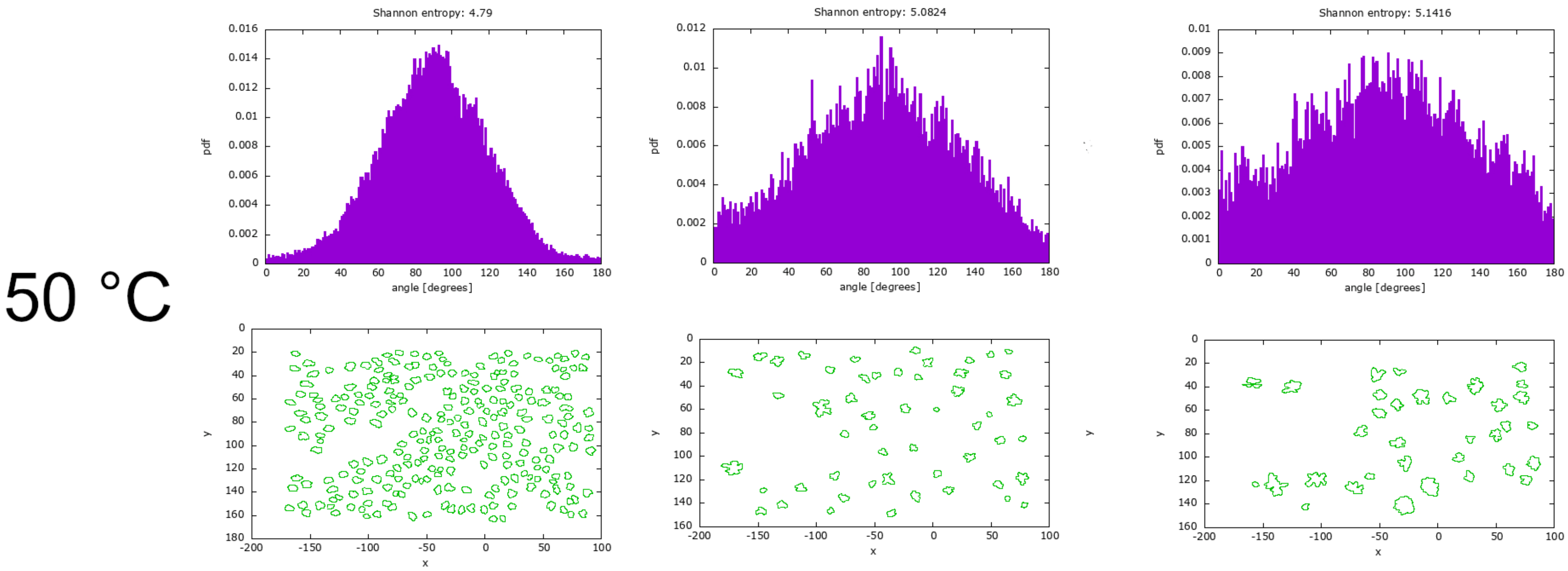

Figure S7. Some pdfs (upper row plots) related to growth steps with different islands configurations (lower row plots) for 6T films grown at 50 °C. Topographic AFM images of 6T islands with different size and density are converted into contour plots picture (lower row plots) with size x and y in pixels (the ratio $x \cdot y^{-1}$ is kept as original AFM images).

8. Absolute Error on *S*: Illustrative Examples of *S* Variability in the Same AFM Image

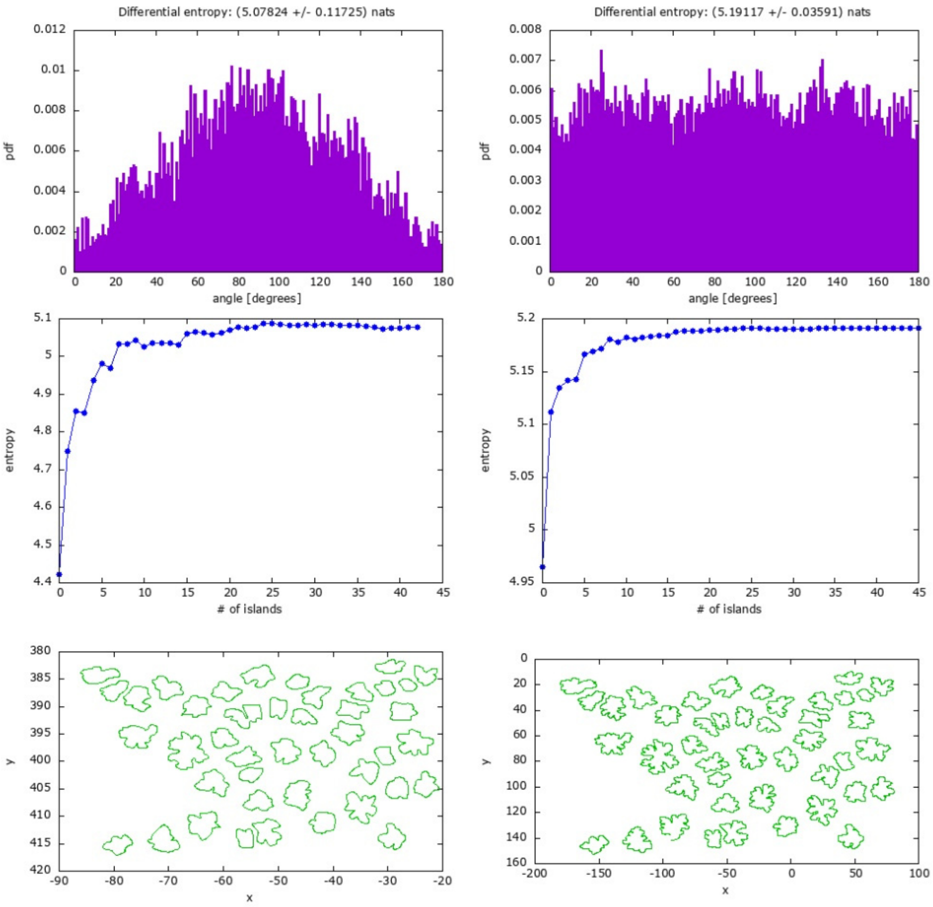

Figure S8. Illustrative examples of – mostly – compact (left) and – completely – dendritic (right) islands affecting the absolute error on *S*. AFM images are processed as described in Sections 3 and 4 (see bottom images of columns) and they have the same number of islands (# in the middle plots), i.e. 45. The processed AFM image on left column shows mostly compact islands with some dendritic ones; this morphology increases the variability of *S* that shifts from ~ 4.4 to ~ 5.1 nats, and thus the absolute error on *S* is larger. Conversely, the processed AFM image on right column shows completely dendritic islands; this homogeneous morphology decreases the variability of *S* that shifts from ~ 4.96 to ~ 5.2 nats thus the absolute error on *S* is smaller.

9. Imaging Quality Parameters Affecting *S* Calculus

AFM images have pixels size of 1024 × 563 px^2 and, after processing, return a two-levels bitmap image where islands -white- were clearly separated from the background–black–. In order to understand *S* trends, some tests on *S* measurements were performed on synthetic images by changing three image quality parameters (surface coverage, islands size, superficial density, image resolution) and/or by using prototypical shapes [12].

In the first test, islands sizes are rescaled of ½ and ¼ with respect to the original size whereas the black background is fixed to 1024 × 563 px^2. This test simulates a surface

coverage decreasing, i.e. islands with constant shape occupy a progressive smaller surface portion (see Figure S9).

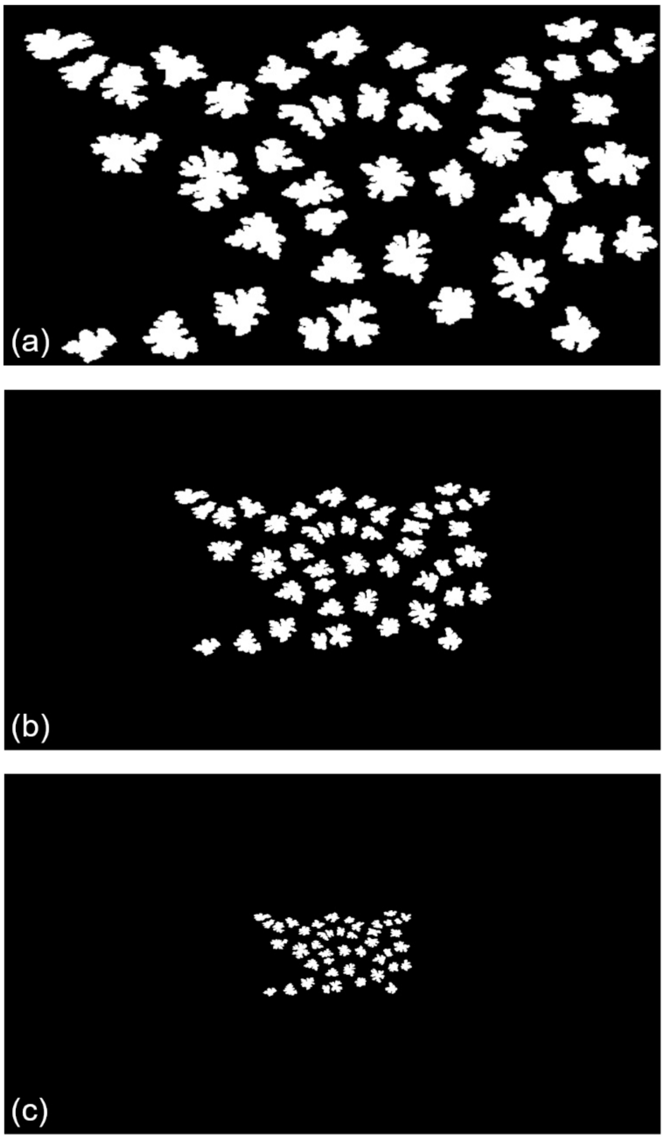

Figure S9. (**a**) The original two-level bitmap image has a density of ~ 2.2 μm^{-2} and islands cover ~ 22% of the surface; (**b**) As islands are rescaled of ½ respect to original size, the surface coverage is reduced to ~ 6%; and to ~ 1.5% by rescaling of ¼ (**c**). The surface density depends on the number of islands therefore is kept constant to ~ 2.2 μm^{-2}.

As clearly shown in Table S1, surface coverage doesn't affect *S* calculus that remains constant within experimental errors. The high value of *S* is determined by the dendritic shape of islands while the progressive increase of its absolute error for decreasing SF is due to the decreased precision of the segmentation process for decreasing islands size.

Table S1. Effect of the Scaling Factor (SF) on the differential entropy *S*.

SF	*S*
/	nats
1	5.19 ± 0.04
½	5.17 ± 0.07
¼	5.08 ± 0.12

In the next test, islands density δ is increased of 4 (~ 8.9 μm^{-2}) and 16 (~ 34.6 μm^{-2}) with respect to the original density (~ 2.2 μm^{-2}) whereas the black background is fixed to 1024 × 563 px^2. This test simulates an island density increasing, i.e. islands with constant shape are progressively more numerous as well as smaller in the same surface area. As shown in Table S2, S is reduced for the highest density because of islands size is reduced and, consequently, its dendric shape is less pronounced, i.e. segmentation process is not able to follow all islands branches due to the size reduction. Indeed, the *quasi*-rectangular pdf typical of dendritic shape is progressively transformed into a Gaussian distribution (not shown) with a constant baseline typical of a compact island (cp. to main text).

Table S2. Effect of the islands density (δ) on the differential entropy S.

$\boldsymbol{\delta}$	$\boldsymbol{S}$
μm^{-2}	nats
2.2	5.19 ± 0.03
8.9	5.18 ± 0.05
34.6	5.06 ± 0.05

The entropy S has been calculated on synthetic images composed of islands with both well-defined (circles and stars) and highly dendritic shapes (Figure S10). Average islands area A_{px} (in px) is measured as the average number of pixels included in each island as selected by the threshold process (cp. to Section 1). The average A_{px} is progressively increased from few tens of pixels to ~ 11,000 px with several area steps (for example, 6 steps were used for describing S vs A_{px} for circles).

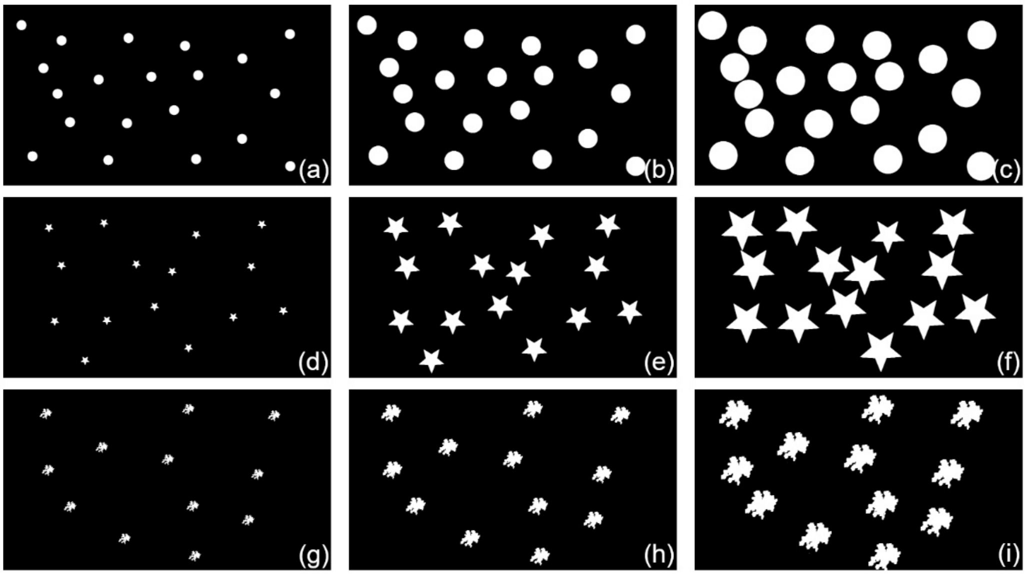

Figure S10. Two-level bitmap of synthetic images with circles (**a-c**), stars (**d-f**) and dendritic islands (**g-i**) with increasing area A_{px} in the following ranges (images in column): 400 – 800 px (**a,d,g**), 1600 – 2800 px (**b,e,h**) and 4500 – 6300 px (**c,f i**).

Entropy measurements are summarized in the plot of Figure S11. As first clear result, S tends to saturate for larger average area A_{px} of islands and their shape determines the average S value: ~ 5.1 nats for dendritic islands, ~ 4.1 nats for star islands and ~ 1.2 nats for circular islands. All S measurements are within the range [0, ~ 5.2] nats, as explained herein in Section 2 and in the main text. Once islands area is small, $A_{px} < 190$ px (left region with respect to the dashed blue line in Figure S11), S varies largely in a random way (stars) or decreasing exponential (circles). Such variations are due to the segmentation process at this scan-size (1024 × 563 px^2, 6.0 × 3.3 μm^2): when islands are too small, the segmentation

process fails to follow islands contours thus, for example, a star became similar to a circle. After these small sizes, S reaches a *plateau* for A_{px} > 190 px (critical area) so S is independent to its average area A_{px} but it depends only on islands contours. This is not strictly true for circular islands because S decreases monotonically following roughly an exponential decreasing. In the pixel range where other shapes saturate (A_{px} > 190 px), S decreases monotonically of ~ 0.6 nats that is, however, lower than the decrease of ~ 2 nats occurring for A_{px} < 190 px.

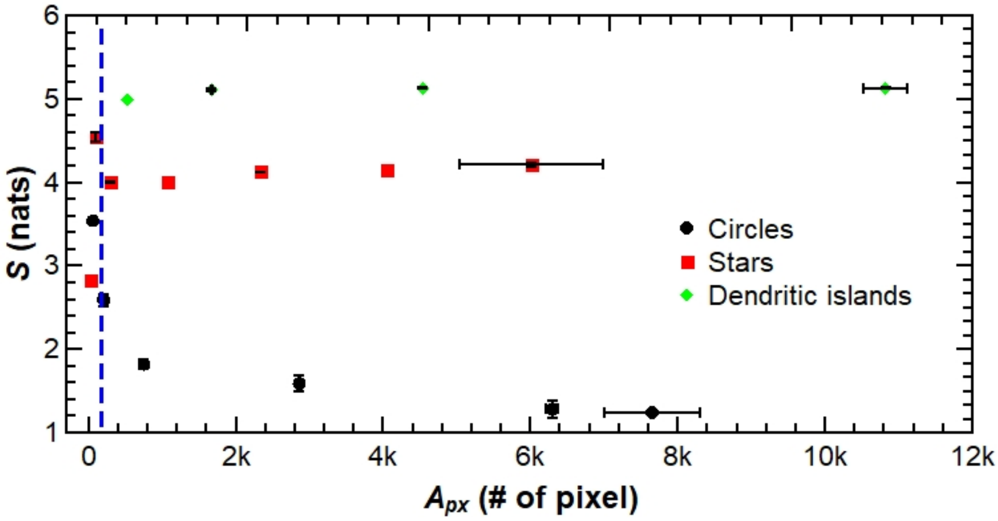

Figure S11. Plot of S (in nats) vs A_{px} (in thousand – k – of pixels, i.e. the average number of pixels # included in each island) for circles, stars and dendritic islands. The dashed blue line indicates the critical area of 190 px.

Star and dendritic islands are instructive in terms of S interpretation because: i) a star well-representing 6T islands at the early stage growth for each ML (see Figure 4 in the main manuscript) with a central body and few (5) branches; ii) synthetic dendritic islands are similar to islands in the next deposition steps of each ML (see Figure 3 and 4 in the main manuscript). The S results obtained with synthetic islands agree with experimental results obtained with 6T islands where S increases from ~ 4.6 nats for star-like islands to the saturation level at ~ 5.2 nats for dendritic-like ones.

Star islands are also important to understand differences between synthetic and real islands. A synthetic star is composed of straight lines for which the segmentation process is very precise as well as its S value (~ 4.1 nats). A real 6T island should has a slightly higher S value because of it is not composed of straight lines. In order to verify this hypothesis, two synthetic images of filled David stars are compared: an image with perfect stars and another one composed of stars with a slightly defective and curved contour (see Figure S12). Islands size is larger than 30 px, granting that S is in the *plateau*. Perfect stars have S of 3.783 nats (with an absolute error at the fifth significant digit) whereas stars with curved and slightly defective contour have S of 4.552 nats with an S increase of the 20%. This test clearly show how S is highly dependent on both shape and defects and/or details of contours, proving that the entropy S is an experimental parameter with a geometrical sensitivity larger than the fractal dimension D_f.

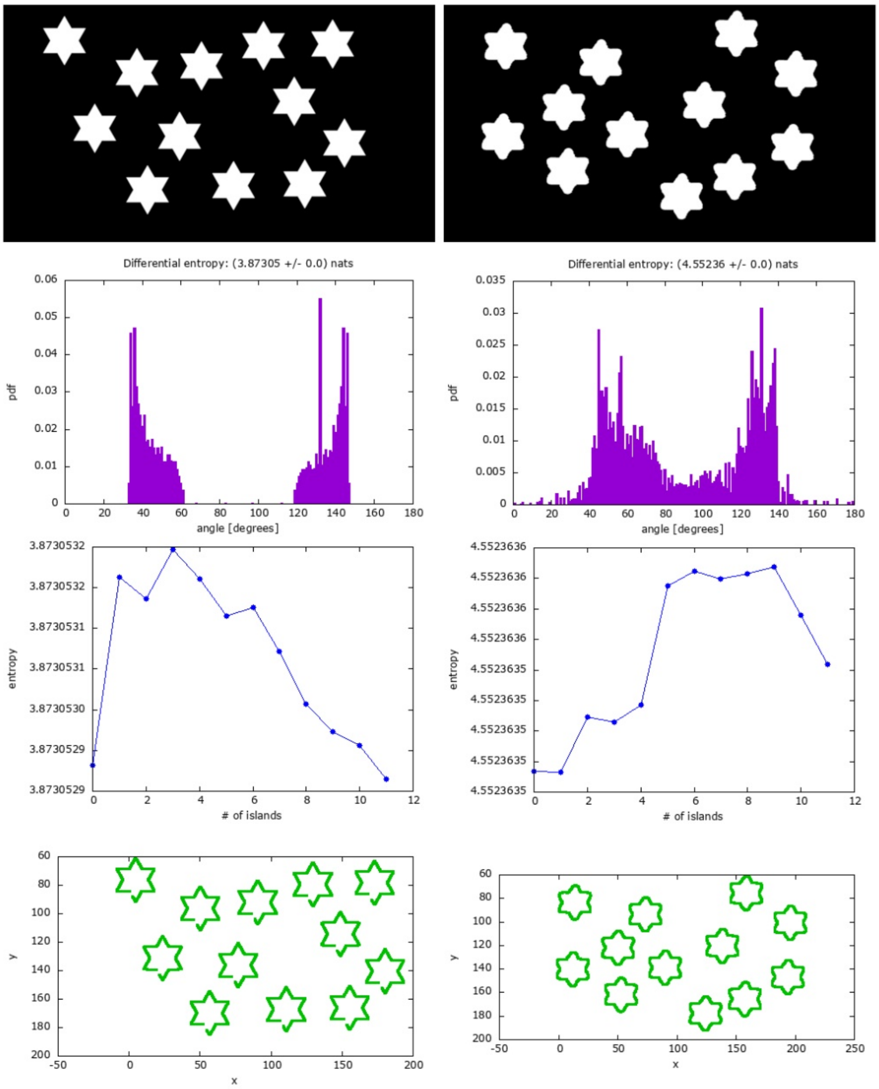

Figure S12. Images and *S* analysis of synthetic filled David stars with perfect (left column) and curved and defective (right column) contours.

Lastly, it is important understand when *S* tends to 0, that is the trivial case when synthetic image is only the black background (no islands are present on the surface). Circular islands are instructive for inferring when *S* tends to 0. A shown in Figure S11, smallest circle has S = 3.54 nats but, inspecting carefully the segmented image obtained for circle with A_{px} ~ 60 px (first point in the plot of Figure S11), the segmentation process depicts squares rather than circles (see Figure S13). A square has a larger *S* than a circle, explaining why the first point of circles has larger *S* than the first point of stars (see Figure S11).

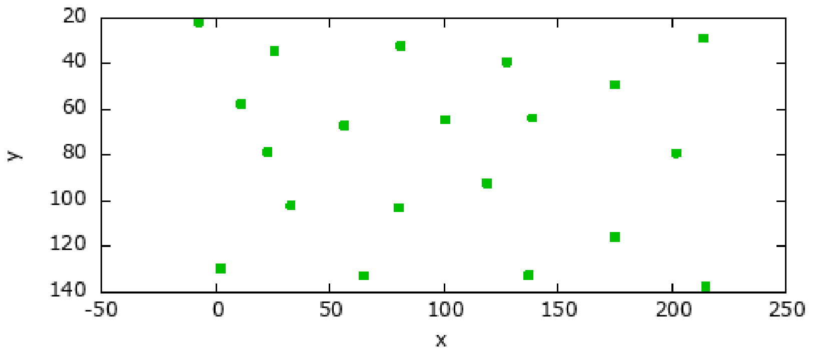

Figure S13. Segmented image for circle with area $A_{px} \approx 60$ px.

From $A_{px} > 190$ px, the circular shape is better defined with a relative error on S that is progressively reduced from 3% to 8‰, for sizes from 750 to 7700 px, respectively.

The exponential decay of S vs circle size suggests that $S \rightarrow 0$ for a size tending to ∞. This result seems counterintuitive, but it is perfectly in agreement with the definition of image resolution (IR) for which, if IR is changed, pixels displayed per inch of the image are changed, not how many pixels are used to make up the image. In the test described above, the IR (herein a pixel density of ~ 171 px·μm^{-1}, i.e. ~ 43 × 10^5 ppi) has been kept constant while the pixel used to make up objects (circle, star and dendritic) with progressively larger size are increased. A circle is poorly defined when it is small (lower number of pixels used to make up, Figure S14a) but its resolution increases when a higher number of pixels are used (Figure S14c). This is particularly true for curved but simple objects (like a circle) where S is affected by the contour resolution, while it is less relevant (except for very small objects at this image size) for regular shapes with straight lines (like stars) and very complex contours (like dendritic islands). Indeed, stars and dendritic islands saturate after 30 px while circle do not. Thus, the paradox of $S \rightarrow 0$ for a size tending to ∞ is resolved.

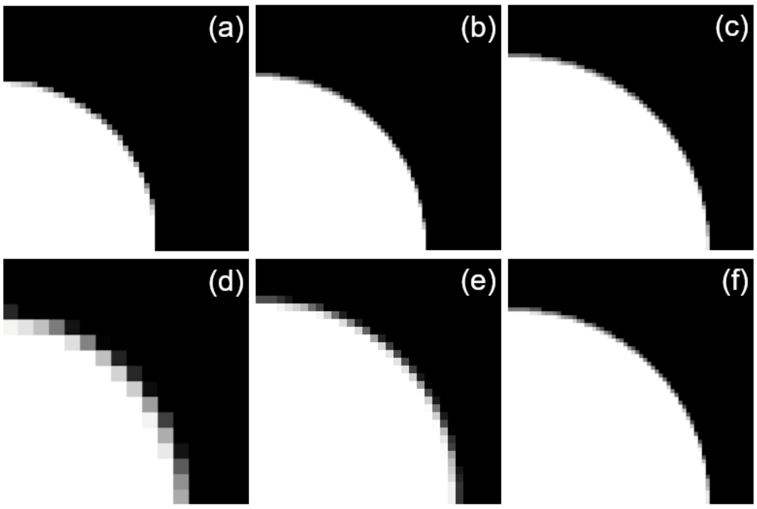

Figure S14. Contour of a circle quadrant for increasing size (60, 90 and 100 px, **a-c**) and for increasing image resolution (256, 512 and 1024 px, **d-f**).

To prove this observational way of thinking, IR of bigger circle (100 px) is decreased to 512 px and 256 px (Figure S14d-f- Our analysis procedure employs a minimized number of Berzier curves to segment objects contours and it works well up to an IR of 1024 px. For higher IR (like 2048 px), the code employs a large number of Berzier curves to describe contours, failing in minimization procedure). Consequently, S value is increased and it

not comparable to other S values). The contour resolution is progressively increased and, accordingly, the entropy S is progressively decreased (see Table S3) because of, at the lowest resolution (Figure S14d) the segmentation process transforms a circle into a polygon.

Table S3. Effect of the image resolution (IR) on the differential entropy S.

IR	S
px	nats
256	2.2 ± 0.1
512	1.66 ± 0.06
1024	1.24 ± 0.01

In conclusion, S tends to 0 if the size of image objects tends to 0 (trivial case) and their shapes tend to a perfect circle (or a perfect polygon). As explained above, this condition cannot be reached by keeping constant the image resolution, i.e. exists a critical size for which S value is incorrect, but smaller objects have to be imaged with higher resolution (zoom in in AFM images). This condition makes S measurement scale dependent, so a sequence of image with increased resolution are needed for having experience of all S value within its values range (from 0 to saturation, cp. Section 2 and the main text).

10. Morphological Effects on 6T Sub-monolayer Films Caused by Geometrical Configurations of UHV Systems

In situ and standard OMBD systems are characterized by two different geometrical configurations of the Organic Material Effusion (OME) cell versus the substrate (see Figure S15). Both *in situ* [13] and standard [14] OMBD systems have crucibles for OME cells with comparable diameter (~ 1 cm), granting the same exiting molecular flux.

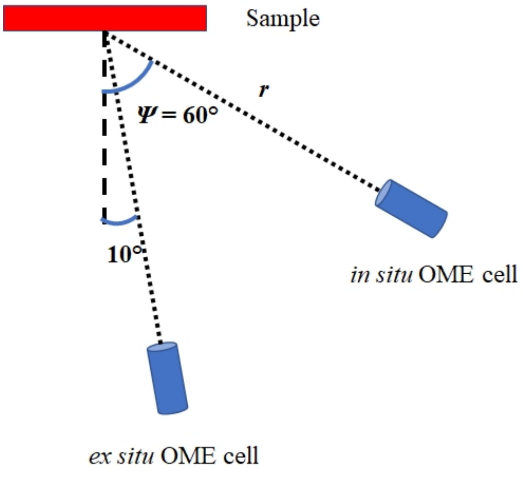

Figure S15. Geometrical schemes of KCs vs substrate for in situ and ex situ OMBD systems.

Understanding the effect of the geometrical configuration of the OMBD system on the morphology of 6T sub-monolayer films is fundamental to explain the higher islands density in *in situ* deposition. The equation describing the material flux $\mathcal{R}$ deposited on a substrate and attached vertically above the OME cell at a distance r is [15]:

$$\mathcal{R} = \mathcal{R}_0 \frac{\cos^4 \psi}{r^2} \quad \text{(S11)}$$

where $\mathcal{R}_0$ is the ideal vapor flux when the OME cell is orthogonal with respect to the substrate plane, r is the distance between the radial center of the OME cell and the center

of the substrate and ψ is the angle between the unit vector normal to the substrate plane and the OME cell (see Figure S15).

The *in situ* OMBD system is characterized by $r = 9$ cm and $\psi = 60°$ whereas a standard OMBD system is characterized by $r = 9$ cm (intentionally fixed equal to the *in situ* system for evaluating solely the effect of the inclination angle) and $\psi = 12°$ (as determined by the standard flange multiport DN100CF – DN40CF, e.g. DN35CF-DN40CF (2.75" OD) 3-Fxd Thru Bolt Holes from the Kurt J. Lesker Company® [16]). By following Equation S11 returns:

$$\mathcal{R}_{in} = \mathcal{R}_{0-in}\frac{\cos^4\psi_{in}}{r_{in}^2} = \mathcal{R}_{0-in}\frac{0.5^4}{81} = \mathcal{R}_{0-in}0.77\cdot 10^{-3}$$
$$\mathcal{R}_{st} = \mathcal{R}_{0-st}\frac{\cos^4\psi_{st}}{r_{st}^2} = \mathcal{R}_{0-st}\frac{0.978^4}{81} = \mathcal{R}_{0-st}11\cdot 10^{-3} \quad \text{(S12)}$$

Fixing the same deposition rate, i.e. $\mathcal{R}_{in}$ and $\mathcal{R}_{st}$ equal to 1 Å·min^{-1}, the ratio between them is:

$$\frac{\mathcal{R}_{in}}{\mathcal{R}_{st}} = \frac{\mathcal{R}_{0-in}0.77\cdot 10^{-3}}{\mathcal{R}_{0-st}11\cdot 10^{-3}} \Rightarrow 1 \approx \frac{1}{10}\frac{\mathcal{R}_{0-in}}{\mathcal{R}_{0-st}} \Rightarrow \mathcal{R}_{0-in} \approx 10\mathcal{R}_{0-st} \quad \text{(S13)}$$

As proved in Equation S13, the ideal vapor flux $\mathcal{R}_{in}$ is ten times faster than $\mathcal{R}_{st}$, explaining why the superficial islands density is high.

References

1. Nečas, D.; Klapetek, P. Gwyddion: an open-source software for SPM data analysis. *Cent. Eur. J. Phys.* **2012**, *10*, 181–188, doi:10.2478/s11534-011-0096-2.
2. Schneider, C.A.; Rasband, W.S.; Eliceiri, K.W. NIH Image to ImageJ: 25 years of image analysis. *Nat. Methods* **2012**, *9*, 671–675, doi:10.1038/nmeth.2089.
3. Shannon, C.E. A mathematical theory of communication. *Bell Syst. Tech. J.* **1948**, *27*, 379–423, doi:10.1002/j.1538-7305.1948.tb01338.x.
4. Marsh, C. Introduction to continuous entropy. Dep. Comput. Sci. Princet. Univ. **2013.** https://www.crmarsh.com/static/pdf/Charles_Marsh_Continuous_Entropy.pdf, accessed on 25 August 2021
5. Godden, J.W.; Bajorath, J. Differential Shannon Entropy as a Sensitive Measure of Differences in Database Variability of Molecular Descriptors. *J. Chem. Inf. Comput. Sci.* **2001**, *41*, 1060–1066, doi:10.1021/ci0102867.
6. Michalowicz, J.V.; Nichols, J.M.; Bucholtz, F. Calculation of Differential Entropy for a Mixed Gaussian Distribution. *Entropy* **2008,** 10.
7. Wikipedia contributors Image segmentation - Wikipedia, The Free Encyclopedia 2021. https://en.wikipedia.org/wiki/Image_segmentation: accessed on 18 October 2021
8. Inkscape Project Inkscape. https://inkscape.org/release/inkscape-1.1.1/ accessed on 21 October 2021
9. MDN Contributors Paths Available online: https://developer.mozilla.org/en-US/docs/Web/SVG/Tutorial/Paths: accessed on 23 September 2021
10. Wikipedia contributors Bézier curve - Wikipedia, The Free Encyclopedia 2021**.** https://en.wikipedia.org/wiki/B%C3%A9zier_curve#Higher-order_curves: accessed on 21 October 2021
11. Wikipedia contributors Centroid - Wikipedia, The Free Encyclopedia 2021. https://en.wikipedia.org/wiki/Centroid#Of_a_polygon: accessed on 24 September 2021.
12. Nečas, D.; Klapetek, P. Synthetic Data in Quantitative Scanning Probe Microscopy. *Nanomater.* **2021**, 11.
13. Dr. Eberl MBE-Komponenten GmbH OME- Organic Material Effusion Cell Available online: https://www.mbe-komponenten.de/products/mbe-components/effusion-cells/ome.php: accessed on 23 September 2021.
14. Murgia, M. Realization of a single filament temperature gradient effusion cell 2004 Priority number BO2000A-000272 on 15/05/2000 PCT/EP 01321252 on 8/01/2004.accessed on 21 October 2021
15. Drechsel, J.; Fröb, H. Deposition of Functional Organic Thin Layers by Means of Vacuum Evaporation. *Vak. Forsch. und Prax.* **2008**, *20*, 15–20, doi:https://doi.org/10.1002/vipr.200890034.
16. Kurt, J. Lesker Company® DN35CF-DN40CF (2.75" OD) 3-Fxd Thru Bolt Holes Available online: https://www.lesker.com/newweb/images/product_drawings/dwg-hd-cf0600a3.svg: accessed on 23 September 2021.
